# Work ability of Thai older Workers in Southern Thailand: a comparison of formal and informal sectors

**DOI:** 10.1186/s12889-021-10974-8

**Published:** 2021-06-24

**Authors:** Sasithorn Thanapop, Chamnong Thanapop

**Affiliations:** 1 The Department of Environmental Health and Technology, School of Public Health, Walailak University, Nakhon Si Thammarat, Thailand; 2 Research Center of Workers Health, Walailak University, Nakhon Si Thammarat, Thailand; 3 The Department of Community Public Health, School of Public Health, Walailak University, Nakhon Si Thammarat, Thailand

**Keywords:** Work ability, Older workers, Formal and informal sectors

## Abstract

**Background:**

Thai society is becoming an ageing society. Independent older persons need to be able to continue to work after retirement. The Work Ability Index (WAI) is an assessment tool for improving the health and work environment of the older workers. The objective of this study is to explore work ability and its related factors among older workers in formal and informal sectors in southern Thailand.

**Methods:**

This cross-sectional study with multistage sampling focused on 324 Thai older workers, aged between 45 and 70 years, working in Nakhon Si Thammarat province. Data on sociodemographic status, health history, and work-related factor questionnaires were collected, including anthropometric measures and the WAI instrument between March and September 2019. Descriptive and logistic regression analyses were used to examine associations.

**Results:**

The participants were predominantly general labourers (23.8%) and female (70.7%). Nearly half of them had noncommunicable chronic diseases (NCDs) (48.2%) and were obese (more than 60%). Approximately 60% (59.9%) engaged in safe working practices. The participants sometimes received occupational health services (51.9%) and frequently accessed health promotion services (78.1%). There was a significant difference in the total average WAI score of the formal and informal workers: 40.6 (S.D. = 4.6) and 37.5 (S.D. = 5.0), respectively. The multivariate analysis showed that workers aged 55 years and older (adj. OR = 1.45; 95% CI [1.21, 1.74]), those with NCDs (adj. OR = 2.85; 95% CI [1.69, 4.80]), and those who were exposed to unsafe working practices (adj. OR = 2.11; 95% CI [1.26, 3.55]) had a higher risk of a poor to moderate WAI.

**Conclusions:**

Most of the older workers had good to excellent work ability. Older age and the presence of NCDs were negatively associated with good to excellent work ability. Safe working practices improved older workers’ work ability. Integrated occupational health protections and health promotion programmes for older informal workers should be provided by community health services to improve work ability.

## Background

Thai society is moving towards becoming an ageing society. The population aged 60 years and over has been slowly increasing. In 2019, older people comprised 17.5% of the nation’s population, which is considered an ageing society, as defined by the United Nations [1, 2]. Thai society will become a completely aged society when the proportion of older people increases to approximately 20%, which is expected to happen in 2022 [1]. The number of older workers has increased yearly, from 3.10 million people in 2010 to 4.70 million in 2020 [3]. The World Health Organisation (WHO) and European countries define older workers as workers aged 45 years and over [4]. The International Labour Organisation (ILO) describes older workers as those who are beginning to have difficulty with their employment and/or occupation due to increasing age [5]. Thailand has identified older workers as those aged 60 years and older who are still working and able to perform their duties [3]. However, based on physical deterioration or physiological changes that affect an individual’s working potential, some people may be considered older workers at the age of 45 years.

The 2nd National Plan for the Elderly 2002–2021 indicated that 67% of independent older people need to continue to work after retirement [6]. The main reasons for working in old age are the workers’ health and his or her family income [7]. Evidence suggests that older people will remain in the workforce for longer periods in response to several social and economic drivers [8, 9]. The vast majority of Japanese workers aged 60 years and over remain in the labour force to maintain their standard of living and for economic reasons. Five factors contribute to the differences in the labour force participation rates of older workers in the United States and Japan: 1) economic need; 2) type of employment; 3) cultural values; 4) policy factors; and 5) health [10]. In Thailand, informal workers continue to work in old age to generate income and security because unlike civil servants or formally employed workers, informal workers do not receive a pension. Legally, informal workers do not have the right to receive the same benefits as formal workers [11, 12].

Older workers’ productivity depends on their health and work-related health, which can be compromised by injuries due to long-term strain, backaches, hearing loss, eye strain, and mental illness. Moreover, ageing- and work-related conditions—such as disease history, behavioural health, work hazards and health care access— are two main factors that threaten their work ability and can cause decreases in work opportunities, productivity and income stability [13].

The increasing trend of older workers in the community thus presents significant issues for worker health. The health needs of older workers comprise physical function, fitness and the working ability needed to perform any job. The Work Ability Index (WAI) is an instrument used in clinical occupational health and research to assess working ability during health examinations and workplace surveys. The index comprises work demands and the worker’s health status and resources, making it an appropriate tool for assessing older worker’s health needs [14]. The assessment results can help community occupational health services to support older workers’ health.

A number of studies have been conducted among different populations to determine the relation between individual and work-related factors and the WAI [15–18]. However, the WAI has not been used for comparative studies of informal and formal older workers in Thailand. The differences between these two groups in terms of socioeconomic and work-related factors, health status, healthcare experience, and WAI have not been studied. Nonetheless, there is a need to improve work ability given the expected increase in the number of older workers in the near future. This study explored work ability and its related factors among Thai older workers in formal and informal sectors in southern Thailand. To achieve this objective, a survey was conducted in Nakhon Si Thammarat province, the southern Thailand province with the largest population. The study’s findings can be used to support occupational health protection and health promotion programmes that can increase the work ability of older workers by extending their working years and improving their well-being.

## Methods

### Study design and setting

This study employed a cross-sectional design based on quantitative approaches. We conducted surveys in rural and suburban areas of two districts located in Nakhon Si Thammarat province, which has the largest population of older workers among Thailand’s southern provinces. The participants were Thai older workers in the formal (public and private) and informal sectors who were between 45 and 70 years of age and had worked in Nakhon Si Thammarat province for at least 2 y.

### Sample size determination and sampling

The data were obtained from the community health surveys of each sub-district. The study population was 112,117, which was calculated by the finite population proportion formula [19] with *p* = 0.28 (proportion of people aged between 45 and 70 years in the population of Nakhon Si Thammarat province); adjustment to ensure an extra 5% yielded a minimum sample size of 324. Community areas and occupation types were submitted to multistage sampling proportional to the selected size. First, the study area was selected; the two districts with the largest older populations were chosen. Second, stratified random sampling was performed by dividing the older workers into two strata: Formal and informal workers. We considered the topography of the sub-districts and villages accordingly and then determined the sub-strata’s proportional sample size for each stratum. Finally, we used simple random sampling of organisational name lists to select formal workers and community health centre registries to select informal workers.

### Data collection and measurements

Data were collected through personal, face-to-face interviews conducted by four trained data collectors between March and September 2019. Data quality was controlled in the field by supervisors from the School of Public Health, Walailak University, and investigators.

### Sociodemographic status, health history and work-related factors

To evaluate study factors, we used a separate questionnaire designed by the researchers. Sociodemographic factors included worker sectors, gender, age, marital status and educational level; health-related factors included noncommunicable chronic diseases (NCDs), smoking status, alcohol consumption and regular exercise activity. The work-related factors in this study were divided into two categories: Work environment and psychosocial exposures [13, 20, 21]. The work environment comprised physical hazards (heat, insufficient light, noise), chemical hazards (pesticide, gas or vapours, dust), biological hazards (poisonous animals), and biomechanical hazards (repetitive work motions, sitting or standing for a long time, twisting and bending the body, lifting or moving heavy objects). The psychosocial exposures comprised unstable income, fast work pace, work overload and work-related stress. In addition, we collected data on work practices, occupational health service experiences (health education, occupational health risk and working process assessments, and primary diagnosis), health promotion service experiences and the utilisation of primary care (health education and counselling, primary prevention programme and NCD screening). Two measurements were used to assess the overall quality of the questionnaire. The index of item objective congruence (IOC) yielded a value of 0.9–1.0 for content validity, and the Cronbach’s alpha coefficient was 0.86 for reliability.

### Measurements

Anthropometric data (weight, height and waist circumference (WC)) were collected using a standardised digital scale (TANITA UM-070, TANITA Corporation, Japan) and a standard measuring tape to the nearest 0.1 kg and 0.1 cm, respectively. Measurements were taken with the participants wearing light clothing without shoes. WC was measured against the participants’ bare skin on a horizontal plane midway between the inferior margin of the last rib and the iliac crest [22]. Obesity was determined through the calculation of body mass index (BMI) and WC for Asians; BMI > =23 kg/m^2^ was used to define overweight, and > =25 kg/m^2^ was used to define obesity; WCs > 90 and > 80 cm was used to define abdominal obesity in men and women, respectively [22–24].

### Work ability index (WAI)

The WAI was developed by the Finnish Institute of Occupational Health Research. This index is aimed at assessing work ability during health examinations and workplace surveys and preventing early retirement and work-related disability [20]. The WAI is calculated by summing the scores of its seven items (range 7–49). The resulting score is used to classify work ability into one of four categories: Poor (7–27), moderate (28–36), good (37–43) and excellent (44–49) [14]. We used the WAI questionnaire version translated in Thai by Kaewboonchoo (2015) for the assessment [25]. However, to analyse the impact of independent variables on different domains of the WAI, we combined the seven items of WAI into three domains according to the purpose of WAI, as has been done in other researches [13, 14, 26]. These domain classifications considered work demands as well as the following: 1) perceived work ability—items 1, 2 and 6); 2) worker health status—items 3, 4 and 5); and mental resources—item 7.

### Study variables

In this article, the dependent variables were the WAI and each work ability domain: Perceived work ability, health status, and mental resources. The explanatory variables included sociodemographic factors, health factors and work-related factors.

### Statistical method

Both descriptive and inferential statistics were performed using R 3.2.1 for Windows (CRAN, 2016). The proportions of the variable of interest explained by sociodemographic, health factors and work sectors were calculated. Independent t-tests and Pearson’s chi-squared tests were used to examine the dimensions of work ability, total WAI score, and the work ability domain for workers in the different sectors. Logistic regression was performed to analyse the determinants of the WAI. We categorised the WAI into two levels: 36 or less (the “poor” and “moderate” classes) and above 36 (“good” and “excellent”), which was the reference category [27]. The three WAI domains were categorised as low or high (“weak” or “strong” and “bad” or “good”) by using the median of the sum of the scores [13]. Independent variables were included in the logistic regression model according to their significance in the bivariable analysis (a *p*-value less than or equal to 0.2) and their lack of collinearity. Therefore, work sector, age group, NCDs, WC, BMI, work practices, and occupational health services experiences were used as the controlled variables in the model. The independent variables were tested in the model using the backward selection method. The final results were considered significant at the 5% significance level (*p* < 0.05).

## Results

### Characteristics and health factors

Table 1 presents the distribution of individual health status, behaviour, and occupational health variables within the sample. The participants (*n* = 324) were predominantly general labourers (23.8%), female (70.7%), and 60–70 years old (37.4%), with an average age of 56.2 (S.D. = 7.4); most were married (74.7%) and had a primary school education (60.8%). Nearly half of them had NCDs (48.2%). More than 70% were never-smokers and never-drinkers, and 59% did not exercise regularly. Their current work duration was less than 10 years (42.6%), and their income was less than 5000 Thai baht (38.9%). The rate of obesity determined by WC (63.6%) was similar to the rate of overweight (65.4%) determined by BMI. Approximately 50% of the participants were exposed to mild or moderate hazards in the work environment, and nearly 60% engaged in safe working practices. Most of the participants sometimes received occupational health services (51.9%) and frequently received in health promotion services (78.1%). The study participants who worked in the informal sector (78.1%) most often had a primary school education (71.1%), had a mean age of 57.4 years, had NCDs (51.8%), and had an income ≤10,000 Thai baht per month (79.1%). They were exposed to a moderate to high level of hazards in the work environment (51.8%), mildly exposed to psychosocial factors (58.9%), generally engaged in safe working practices (57.3%), sometimes had access to occupational health services (54.2%), and frequently had access to health promotion services (77.1%). On the other hand, the formal workers educated at secondary school or higher level (74.6%), mean age was 51.9, had NCDs (35.2%), income > 10,000 Thai baht per month (61.9%). More than half of them were exposed to the work environment and psychosocial factors at a mild level, safe working practices (69.0%), and frequent access to occupational health services (56.3%) and health promotion services (81.7%).

**Table 1:** Characteristics and health factors of older workers (*n* = 324)

Characteristics	Worker sector: ***n*** (%)		
	formal (*n* = 71)	informal (*n* = 253)	total
**Gender**			
Male	37 (52.1)	58 (22.9)	95 (29.3)
Female	34 (47.9)	195 (77.1)	229 (70.7)
**Age group (y**)			
45–49	25 (35.2)	49 (19.4)	74 (22.8)
50–54	25 (35.2)	55 (21.7)	80 (24.7)
55–59	17 (23.9)	32 (12.6)	49 (15.1)
60*–*70	4 (5.6)	117 (46.2)	121 (37.4)
Mean (S.D.)	51.9 (5.1)	57.4 (7.5)	56.2 (7.4)
Min, Max	45, 67	45, 70	45, 70
**Marital status**			
Single	5 (7.0)	21 (8.3)	26 (8.0)
Married	61 (85.9)	181 (71.5)	242 (74.7)
Widow/ Separate	5 (7.0)	51 (20.2)	56 (17.3)
**Educational status**			
Illiteracy	1 (1.4)	3 (1.2)	4 (1.2)
Primary school	17 (23.9)	180 (71.1)	197 (60.8)
Secondary school	28 (39.4)	51 (20.2)	79 (24.4)
Bachelor degree or higher	25 (35.2)	19 (7.5)	44 (13.6)
**NCDs**			
Absence	46 (64.8)	122 (48.2)	168 (51.9)
Presence (repeated answer)	25 (35.2)	131 (51.8)	156 (48.2)
Hypertension	6 (8.5)	59 (23.3)	65 (20.1)
Dyslipidemia	9 (12.7)	21 (8.3)	30 (9.3)
Diabetes	5 (7.0)	10 (4.0)	15 (4.6)
Chronic respiratory diseases	0 (0)	14 (5.5)	14 (4.3)
others	5 (7.0)	27 (10.7)	32 (9.9)
**Smoking status**			
Never smoker	42 (59.2)	208 (82.2)	250 (77.2)
Former smoker	10 (14.1)	13 (5.1)	23 (7.1)
Smoker	19 (26.8)	32 (12.6)	51 (15.7)
**Alcohol consumption**			
Never drinker	40 (56.3)	201 (79.4)	241 (74.4)
Ever drinker	0 (0)	10 (4.0)	10 (3.1)
Drinker	31 (43.7)	42 (16.6)	73 (22.5)
**Occupation**			
Government employee	30 (42.3)	0 (0)	30 (9.3)
Company employee	26 (36.6)	0 (0)	26 (8.0)
General labour	10 (14.1)	67 (26.5)	77 (23.8)
Rubber plantation	0 (0)	68 (26.9)	68 (21.0)
Fruit plantation	0 (0)	15 (5.9)	15 (4.6)
Merchant	0 (0)	64 (25.3)	64 (19.8)
Personal business	0 (0)	16 (6.3)	16 (4.9)
Others	5 (7.0)	23 (9.1)	28 (8.6)
**Current working duration (y)**			
< 10	32 (45.1)	106 (41.9)	138 (42.6)
10–20	21 (29.6)	65 (25.7)	86 (26.5)
> 20	18 (25.4)	82 (32.4)	100 (30.9)
Income per month (Thai baht)*			
< 5000	9 (12.7)	117 (46.2)	126 (38.9)
5000 – 10,000	18 (25.4)	83 (32.8)	101 (31.2)
10,001 – 15,000	16 (22.5)	21 (8.3)	37 (11.4)
> 15,000	28 (39.4)	32 (12.6)	60 (18.5)
**Regular exercise Activity**			
Yes	33 (46.5)	100 (39.5)	133 (41.0)
No	38 (53.5)	153 (60.5)	191 (59.0)
**Waist circumference**			
Normal	33 (46.5)	85 (33.6)	118 (36.4)
Obesity	38 (53.5)	168 (66.4)	206 (63.6)
**BMI (kg/m**^**2**^**)**			
< =22.9 (normal)	28 (39.4)	84 (33.2)	112 (34.6)
> =23.0 (overweight)	43 (60.6)	169 (66.8)	212 (65.4)
**Work environment exposure**			
Mild	40 (56.3)	122 (48.2)	162 (50.0)
Moderate - high	31 (43.7)	131 (51.8)	162 (50.0)
**Psychosocial exposure**			
Mild	47 (66.2)	149 (58.9)	196 (60.5)
Moderate - high	24 (33.8)	104 (41.1)	128 (39.5)
**Work practices**			
Unsafe	22 (31.0)	108 (42.7)	130 (40.1)
Safe	49 (69.0)	145 (57.3)	194 (59.9)
**Occupational health services experience**			
Sometimes	31 (43.7)	137 (54.2)	168 (51.9)
Frequently	40 (56.3)	116 (45.8)	156 (48.1)
**Health promotion services experience**			
Sometimes	13 (18.3)	58 (22.9)	71 (21.9)
Frequently	58 (81.7)	195 (77.1)	253 (78.1)

Note: 1 USD = 30.1568 Thai-baht (accessed on 18 January 2021)

### Working ability of older workers: dimension, class, and domain

Table 2 shows that the average WAI was 40.6 (S.D. = 4.6) for formal workers and 37.5 (S.D = 5.0) for informal workers. Older workers in the formal sector tended to have a higher WAI scores in every dimension than informal workers did. Independent t-tests showed a statistically significant difference in the WAI scores for each dimension between workers in the formal and informal sectors (*p* < 0.05). Pearson’s chi-square was used to examine the rate difference of WAI classes between formal and informal workers (*p* = 0.002). Most of the workers had a high proportion of good to excellent WAI scores: 81.7% for formal workers and 62.5% for informal workers. We explored the WAIs of workers by sector in the three domains. The analysis found rate differences between WAI categories and work sector in perceived working ability and health status (*p* < 0.001). Among the formal workers, 64.8% indicated strong perceived working ability, while 65.6% of the informal workers reported poor health. Moreover, the formal workers had higher rates of good health than bad health (56.3% vs 43.7%), in contrast with the informal workers (34.4% vs 65.6%). However, there was no difference in mental resources between the two groups of workers.

**Table 2:** Dimension, score, class and domain of WAI of older workers

Items	Formal (***n*** = 71)	Informal (***n*** = 253)	***p***
**Dimension of work ability:** mean (SD)			
1) Current work ability compared with the lifetime best	7.9 (1.4)	7.5 (1.6)	0.016^a^
2) Work ability in relation to the demands of the job	8.7 (1.4)	7.8 (1.6)	< 0.001^a^
3) Numbers of current diseases diagnosed by a physician	5.7 (1.7)	5.3 (1.7)	0.028^a^
4) Estimated work impairment due to diseases	4.5 (1.4)	4.1 (1.8)	0.022^a^
5) Sick leave during the past year (12 months)	4.6 (0.6)	4.5 (0.8)	0.019^a^
6) Personal prognosis of work ability 2 years from now	5.6 (1.6)	5.1 (1.6)	0.004^a^
7) Mental resources	3.4 (0.6)	3.3 (0.7)	0.023^a^
Total work ability index score: mean (SD)	40.6 (4.6)	37.5 (5.0)	< 0.001^a^
Class of work ability: *n* (%)			
Good - Excellent	58 (81.7)	158 (62.5)	0.002^b^
Poor - Moderate	13 (18.3)	95 (37.5)	
Three domains according to the purpose of WAI: *n* (%)			
Perception of work ability			< 0.001^b^
Weak	25 (35.2)	145 (57.3)	
Strong	46 (64.8)	108 (42.7)	
Health status			< 0.001^b^
Bad	31 (43.7)	166 (65.6)	
good	40 (56.3)	87 (34.4)	
Mental resources			0.228^b^
Weak	35 (49.3)	158 (62.5)	
Strong	36 (50.7)	195 (37.5)	

^a^ Independent t-test, ^b^ Pearson’s chi-squared test

### WAI category by Sociodemographic, work-related and health factors

Table 3 shows the bivariate analysis of the rate of poor to moderate versus good to excellent WAI scores according to individual and occupational health factors. The individual factors associated with the WAI category were worker sector, age group, NCDs, WC, and work practices (*p* < 0.05).

**Table 3:** The class of WAI according to sociodemographic, health status and work-related factors

	Poor – Moderate (***n*** = 108)	Good – Excellent (***n*** = 216)	***p***
**Worker sector**			0.002
Formal	13 (18.3)	58 (81.7)	
Informal	95 (37.5)	158 (62.5)	
**Residence**			0.635
Rural	46 (31.9)	98 (68.1)	
Sub-urban	62 (34.4)	118 (65.6)	
**Gender**			0.931
Male	32 (33.7)	63 (66.3)	
Female	76 (33.2)	153 (66.8)	
**Age (y)**			< 0.001
< 55	29 (18.8)	125 (81.2)	
> =55	79 (46.5)	91 (53.5)	
**Marital status**			0.651
Single/widow/ separate	29 (35.4)	53 (64.6)	
Married	79 (32.6)	163 (67.4)	
**NCDs presence**			< 0.001
Absence	34 (20.2)	134 (79.8)	
Presence	74 (47.4)	82 (52.6)	
**Smoking status**			0.852
Never smoker	84 (33.6)	166 (66.4)	
Former/ Smoker	24 (32.4)	50 (67.6)	
**Alcohol consumption**			0.857
Never drinker	81 (33.6)	160 (66.4)	
Ever/ Drinker	27 (32.5)	56 (67.5)	
**Exercise Activity**			0.299
Yes	40 (30.1)	93 (69.9)	
No	68 (35.6)	123 (64.4)	
**Waist circumference**			0.022
Normal	30 (25.4)	88 (74.6)	
Obesity	78 (37.9)	128 (62.1)	
**BMI**			0.117
Normal	31 (27.7)	81 (72.3)	
Overweight	77 (36.3)	135 (63.7)	
**Work environment exposure**			0.814
Mild	53 (32.7)	109 (67.3)	
Moderate - high	55 (34.0)	107 (66.0)	
**Psychosocial exposure**			0.936
Mild	65 (33.2)	131 (66.8)	
Moderate - high	43 (33.6)	85 (66.4)	
**Work practices**			
Unsafe	57 (43.8)	73 (56.2)	0.001
Safe	51 (26.3)	143 (73.7)	
**Occupational health services experience**			0.099
Sometimes	63 (37.5)	105 (62.5)	
Frequently	45 (28.8)	111 (71.2)	
**Health promotion services experience**			0.924
Sometimes	24 (33.8)	47 (66.2)	
Frequently	84 (33.2)	169 (66.8)	

### Determinants of the WAI and its domains

Table 4 demonstrates the multivariable analysis of the determinants of the WAI and its domains. The analysis highlighted the association of age group, NCDs, and work practices with the WAI and its domains. The probability of having a poor to moderate WAI was higher in 55-year and over age group (adj. OR = 1.45; 95% CI [1.21, 1.74]) in those with NCDs (adj. OR = 2.85; 95% CI [1.69, 4.80]), and those exposed to unsafe work practices (adj. OR = 2.11; 95% CI [1.26, 3.55]). The probability of poor perceived work abilities and weak mental resources was higher in the 55 years and over age group (adj. OR = 1.41; 95% CI [1.21, 1.66], and adj. OR = 1.32; 95% CI [1.13, 1.55], respectively). Moreover, a higher probability of poor health status was found in the 55-year and over age group and in those with NCDs (adj. OR = 1.21; 95% CI [1.02, 1.45] and adj. OR = 6.42; 95% CI [3.76, 10.96], respectively).

**Table 4:** The simple logistic regression model of associations between sociodemographic, work-related and health factors with work ability index and it’s domains among older workers

	OR (95% CI)			
	WAI	Perception of work ability	Health status	Mental resources
Worker Sector (formal#)	1.69 (0.83, 3.45)	1.72 (0.95, 3.09)	1.76 (0.95, 3.27)	1.25 (0.71, 2.20)
Age (< 55#)	1.45 (1.21, 1.74)***	1.41 (1.21, 1.66)***	1.21 (1.02, 1.45)*	1.32 (1.13, 1.55)***
NCDs presence (Absence#)	2.85 (1.69, 4.80)***	1.57 (0.98, 2.53)	6.42 (3.76, 10.96)***	1.21 (0.76, 1.95)
WC (Normal#)	1.38 (0.70, 2.72)	0.97 (0.52, 1.79)	1.28 (0.66, 2.49)	1.36 (0.74, 2.50)
BMI (Normal#)	1.10 (0.56, 2.16)	1.17 (0.63, 2.16)	1.34 (0.69, 2.62)	0.87 (0.47, 1.61)
Work practices (Safe#)	2.11 (1.26, 3.55)**	1.59 (0.98, 2.57)	1.06 (0.62, 1.79)	1.25 (0.78, 2.01)
Occupational health service experience (Frequently#)	1.22 (0.72, 2.08)	1.18 (0.73, 1.91)	0.91 (0.54, 1.54)	1.12 (0.70, 1.79)

Note: *** *p* < 0.001, **p < 0.01, *p < 0.05, # Reference group

## Discussion

This study was conducted mainly to explore the differences in the WAI between formal and informal sector workers and the relationship between sociodemographic, health- and work-related factors and the WAI and its domains. The multivariate analysis showed that age group was associated with the WAI and its domains, including the perceived working ability, health status and mental resources of the study sample. Workers in the oldest age group (55 years and over) were at risk of poor to moderate work ability. The presence of NCDs was also negatively associated with WAI and health status, while safe working practices were positively associated with WAI.

The mean total WAI score of the formal workers was 40.6, while that of the informal workers was 37.5. The overall proportion of workers with good to excellent work ability was 66.7%. A higher proportion of formal workers than informal workers reported good to excellent work ability. Demographic factors were associated with higher WAI scores among the worker groups. The proportions of younger workers (less than 55 years of age), workers without NCDs and safe working practices were higher among the formal workers than among the informal workers. The study of Yingratanasuk (2015) focused on informal older workers aged over 60 years and found that 43.4% had good to excellent work abilities. Most of the workers were general labourers and merchants [16]. Moreover, Rattanapunya (2019) studied work ability among informal older workers aged over 45–80 years and found that 13% had a good to excellent WAI [18]. Most of the workers in that study were home-based workers in urban areas, which differed from our study. The previous studies showed a higher mean age of informal workers than our study did. These factors probably affected the lower proportion of good to excellent WAI scores. In contrast, the study by Kaewboonchoo (2011) of the WAI scores of formal workers in small- and medium-sized enterprises found a mean worker age of 34 years and a mean total WAI score of 40 [15]. Our study had a similar mean total WAI score, although the formal workers in our study had a higher mean age. Our study categorised worker age into two groups: Less than 55 years and 55 years and older. The age group was associated with the WAI and its three domains. The risk of a poor to moderate WAI was 1.45 times higher in participants aged 55 years and older. Age was significantly associated with work ability. The direction of this association is consistent with many studies showing that age is significantly and negatively associated with WAI in various occupational sectors [28–31]

However, our results revealed that gender and working ability were not significantly associated in the multivariable analysis [21, 27]. Two factors must be considered for older workers [32, 33]: The first is the workers’ health, and the second is job productivity and performance. A larger number of older workers implies, for example, an increasing number of people working with minor and major health problems that occur more frequently after 55 years of age [34]. Approximately 52% of the workers in both sectors in this study were older than 55 years, and half had NCDs. Age and the presence of NCDs were the predominant factors affecting older workers’ productivity. The results also indicate that the presence of NCDs has strong and positive association with poor to moderate WAI, especially in the health status domain (adj. OR = 6.42). The causal factors of NCDs are complex and have been described as the interaction of work-related risk factors and lifestyle risk factors [35]. Our data show that hypertensive disease accounted for the highest proportion of NCDs (48.2%), and approximately 60% of the workers were overweight or obese, in line with other reports [20, 21, 36–38]. The rates did not differ between the two sectors. Physical health is one of the intermediate determinants of individual lifestyle. It directly affects workers’ health status and functional activity, which are fundamental factors for work ability [39, 40]. Moreover, more than half of the workers (59%) reported that they did not regularly exercise. Therefore, the increasing risk of NCDs among older workers may be due to a decline in physical capability and increased obesity. These factors have a negative impact on older workers’ work ability. Other risk factors, including smoking and alcohol consumption, are not associated with WAI [36]. Therefore, formal workers must engage in NCD prevention and health promotion activities for adult workers before they reach old age. This approach is promoted by the total worker health (TWH) concept, which is a platform for promoting disease and injury prevention efforts in the workplace by developing innovative methods, techniques and approaches for dealing with occupational safety and health problems [41]. Additionally, primary healthcare centres should coordinate with occupational health protection activities aimed at preventing NCDs among community informal workers before they enter old age [42].

We explored personal hygiene, safety inspections, personal protective equipment (PPE) usage, simple work improvements, rest duration and housekeeping as working conditions. The study results demonstrated that workers who were exposed to unsafe practices had a higher probability of a poor to moderate WAI (adj. OR = 2.11). They also showed that workers in the formal sector were more likely to engage in safe work practices than those in the informal sector. The WAI is composed of four dimensions: Health and functional capacity, occupational competence, attitude and motivation, and working conditions. Working conditions comprise the top floor of the four main dimensions; if the individual’s resources (floors 1–3) are able to support the top floor, the individual’s work ability is good [39, 40]. Tuomi et al. (2001) showed that work environment factors, such as poor ergonomics, a distracting work environment, poor physical climate, tool failure and dissatisfaction work rooms, were strongly associated with poor working ability [20]. On the other hand, improvements in work and tasks, work environments and tools positively influenced work ability [20]. Therefore, our results demonstrate that working conditions are among the crucial occupational health factors that can improve the work ability of older workers. The study also found a difference in working practices between the formal and informal sectors. This difference may be related to legal protections for employments and access to occupational health services. In Thailand, informal workers utilise healthcare services provided by the Universal Health Coverage Scheme (UCS). It provides healthcare for people who have no employment benefits, such as agricultural and home-based workers. However, the services do not include occupational health services, which are necessary to help older informal workers improve their work ability. The improvement of worker health to support work ability is complex and requires a holistic approach, especially for older workers. Occupational health service arrangements for older workers should be initiated at the early phase of physical and mental deterioration, as indicated by a decline in the WAI score. The work ability model is appropriate for an older workforce and is based on the self-assessment of subjective experiences regarding personal resources, work context, and work-life interface [39, 40]. The structure of an individual’s work ability changes throughout his or her life and career [43]. Therefore, the implementation of work ability assessments in occupational health programmes for older workers will provide measures to improve work ability in both workplaces and healthcare services centres [44–46].

There are several limitations of this study. First, the cross-sectional design in which exposure and outcome were measured concurrently does not support causal relationships. In epidemiological studies, an association between exposures and outcomes is causal only if the study’s plausibility is explored. Second, self-reported measurements of the study variables—including working ability and occupational hazards—may lead to recall and information bias. Workers’ awareness of occupational hazards could affect the accuracy of the measurements. The workers in the formal sector are familiar with their work conditions and can recognise occupational hazards in their workplace, while informal workers may not be able to do so. Finally, we focused on the working abilities of older workers residing in suburban and rural communities for reasons of access, and this population may not be representative of workers elsewhere in different settings. Therefore, generalisations of the results beyond the study population should consider this limitation.

## Conclusions and recommendations

In conclusion, this study determined the association between work ability and the determinants, health-related factors, sociodemographic characteristics and work environment of formal and informal older workers. Most of the older workers had good to excellent work ability. Older age group and the presence of NCDs were negatively associated with good to excellent work ability. There was a strong negative association between NCDs and the health status of workers. This result suggests that older workers with poor health due to NCDs require more intensive healthcare if they are to extend their working years. In addition, safe working practices improved older workers’ work ability. The work ability of older workers is a very important background factor if workers are to continue to work throughout their lifespan. According to the concept of TWH, formal workers’ organisations could integrate occupational health protections with NCD prevention for workers beginning in the early stages of ageing. Similarly, primary healthcare programmes for NCD prevention in the community should cooperate with occupational health protection services to promote longer healthy lives among informal workers. For further study, a longitudinal study of various occupations and programme interventions is required to determine and compare the factors associated with improved work ability in formal and informal workers.

## Data Availability

The datasets generated and/or analysed during the current study are not publicly available due to concealment of datasets has been identified in the human research ethics request process. Requests for tools and materials should be addressed to the corresponding author.

## Abbreviations

BMI: Body mass index
NCDs: Non-communicable diseases
OR: Odds ratio
WAI: Work ability index
WC: Waist circumference
